# Improving the Dynamic Characteristics of Body-in-White Structure Using Structural Optimization

**DOI:** 10.1155/2014/190214

**Published:** 2014-07-02

**Authors:** Aizzat S. Yahaya Rashid, Rahizar Ramli, Sallehuddin Mohamed Haris, Anuar Alias

**Affiliations:** ^1^Advanced Computational and Applied Mechanics (ACAM) Group, Centre for Transportation Research (CTR), Faculty of Engineering, Universiti Malaya, 50603 Kuala Lumpur, Malaysia; ^2^Centre for Automotive Research, Faculty of Engineering and Built Environment, Universiti Kebangsaan Malaysia, 43600 Bangi, Selangor, Malaysia; ^3^Proton Professor Office (PPO), Proton Bhd. 40000 Shah Alam, Selangor, Malaysia

## Abstract

The dynamic behavior of a body-in-white (BIW) structure has significant influence on the noise, vibration, and harshness (NVH) and crashworthiness of a car. Therefore, by improving the dynamic characteristics of BIW, problems and failures associated with resonance and fatigue can be prevented. The design objectives attempt to improve the existing torsion and bending modes by using structural optimization subjected to dynamic load without compromising other factors such as mass and stiffness of the structure. The natural frequency of the design was modified by identifying and reinforcing the structure at critical locations. These crucial points are first identified by topology optimization using mass and natural frequencies as the design variables. The individual components obtained from the analysis go through a size optimization step to find their target thickness of the structure. The thickness of affected regions of the components will be modified according to the analysis. The results of both optimization steps suggest several design modifications to achieve the target vibration specifications without compromising the stiffness of the structure. A method of combining both optimization approaches is proposed to improve the design modification process.

## 1. Introduction

The dynamic characteristics of a body-in-white structure of a car are important in the design phase. Initially, for most structures undergoing dynamic loading, it is essential to know the natural frequencies and the corresponding mode shapes [1, 2]. The dynamic behavior of a structure can be predicted by knowing the characteristics. By understanding how a structure would react under certain frequency, a number of improvements can be implemented in a design. In such cases like the BIW, the modification of the body needs to be considered in NVH while also focusing on the ride [3], handling [4], and safety of the user. Failures could occur due to resonance or fatigue which can be avoided by identifying the resonance regions and operating frequency of the structure [5].

There are a number of cases where optimization was used to modify smaller parts of the vehicle such as rocker panels, mounting brackets, pillars, seat frames, suspension rings, and steering knuckles. Large parts such as engine blocks, chassis, and whole car bodies have also been used as the basis for optimization. The effectiveness of a car body as a whole was shown to be improved by replacing the baseline material and optimizing the design of the structure [6]. There was also a method introduced to properly design the hood of a car to safely prevent head injuries of a pedestrian in a vehicle-to-pedestrian collision [7].

In this paper, normal modes analysis was used to calculate the natural frequency and the corresponding mode shape of a BIW design. Free-free boundary condition is used to be consistent between results and the high repeatability. This approach is common in determining the global body stiffness of a structure with modal analysis tests [8]. It is then compared to the operating frequency of the structure depending on the vibration that may be induced by a number of factors such as engine vibrations, road conditions, and suspension system [9].

Structure dynamic modifications are executed by using topology optimization with the mass of the structure as the objective. The mass is minimized as to satisfy one of the objectives to develop a fuel-efficient car. Consequently, the frequency of the first bending and torsion modes was used as constraints to suppress vibration [10]. The design variable for the optimization would be the thickness of the components where the basic design of the body would not be influenced by the alteration. This is important as not to affect other factors contributing to the BIW design such as crashworthiness, components placements, and design space [11]. From the optimization, regions where reinforcement is needed can be identified. These regions may also pose other drawbacks such as manufacturing infeasibility or excessive mass increase which can be alleviated by introducing additional processes.

Size optimization [12] was then utilized to ease the manufacturing constraint of the optimization results. The thickness of the specific components can be entirely changed instead of adding little reinforcing support material onto the original structure. The new thickness not only will improve dynamic characteristics of the structure but also will change the behavior under different conditions. Therefore, the amount of changes introduced in the design must be monitored throughout the process to prevent significant drawbacks due to the modifications.

Previously, most approaches on improving the dynamic characteristics of a structure employed trial-and-error methods either physically or virtually (computer models) based on creativity, previous design, or experience. By using structural optimization, the process can be fully utilized even in the design stage without the need of any fabrication. Previous works such as Wang et al. [13] and Jang et al. [11] involve optimization of the dynamic characteristics of an automobile body by maximizing the first torsion and/or bending modes separately. The improvement in the natural frequency for torsion mode in the latter work [11] also demonstrates the ineffectiveness of the method where it required an additional 10% of the original mass to increase the frequency by 2 Hz. The proposed method employs structural topology optimization by considering both bending and torsion modes, simultaneously followed by a size optimization step. An attempt to maximize a parameter without constraining the other will produce a questionable design. The best practice is to set a target value that will still improve the structure while avoiding overcompensation of the reinforcement. Three other factors are considered in this paper in addition to the dynamic characteristics, the original design of the model, the total mass added after the process, and the manufacturability of the components.

## 2. Modal Analysis

For the modal analysis, real eigenvalue analysis was done using Altair-Hyperworks to find the natural frequencies and the corresponding mode shapes ignoring the damping [14]. The results can be used to predict the dynamic behavior of the structure.

**Figure 1:**
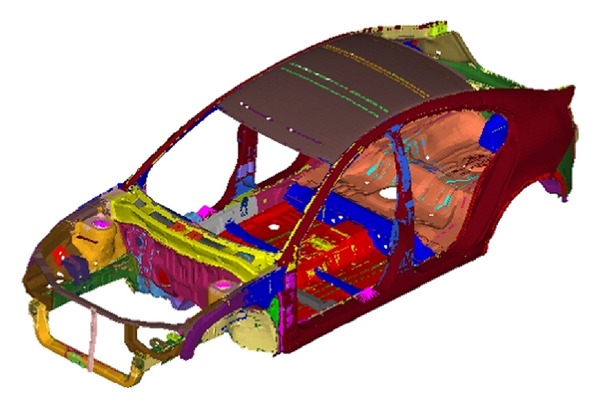
Original BIW model.

**Figure 2:**
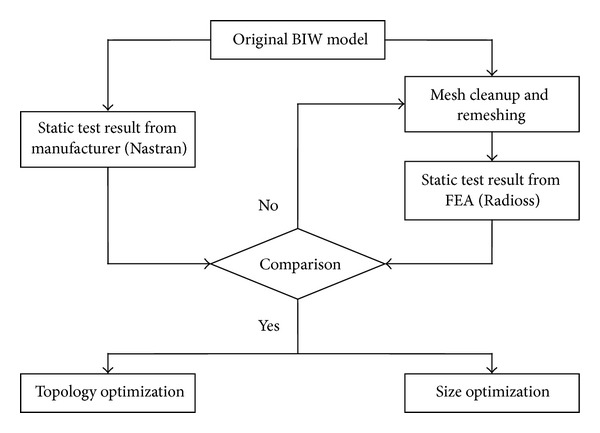
Flow diagram for BIW model optimization strategy.

The model used for this paper is a compact 5-door hatchback with the BIW of the car shown in Figure 1. While the research mainly covers dynamic response of the structure, a static test was also conducted for validation purposes in regard to the other factors. The consistency of the analysis can also be checked, because the dynamic analysis will rely only on the mass and stiffness of the model same as in a simple static analysis.  Figure 2 shows the validation strategy for the model before employing any modification.

**Figure 3:**
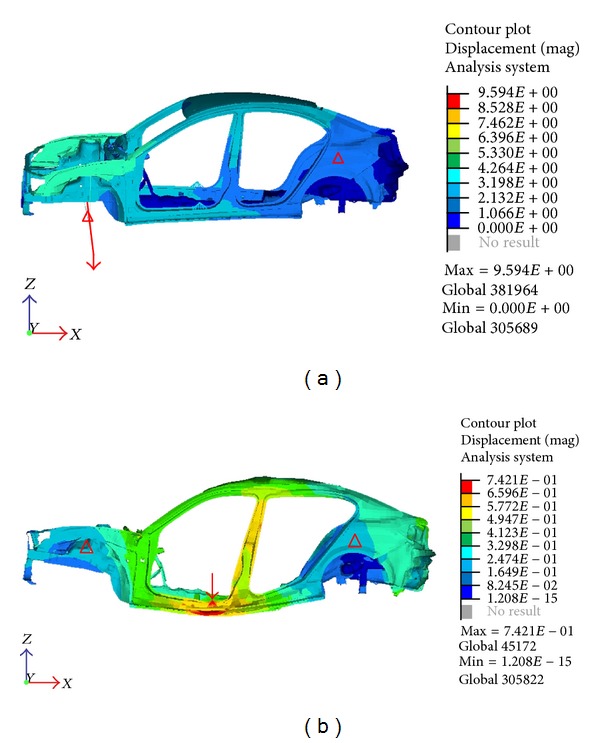
Static torsion (a) and bending (b) analysis.

Static torsion and bending tests were done to verify the stiffness of the body on which the durability of the entire car would depend. The test was conducted by calculating the deflection at specific location of the body when the loads were applied. In both cases, the structure was constrained in all degrees of freedom at the rear wheel axle center line.  Figure 3 shows the result from both static test based on the displacement of each elements. For torsion stiffness test, the center of the front axle was also constrained in the vertical direction. The moment was applied along the front axle with the force far enough to avoid any influence on the deflection result. The position of the wheel base was used as the point of interest in the test. During stiffness test for bending, the front wheel centerline was also constrained in all degrees of freedom except for the horizontal direction. The force was applied at the center base of the quarter panel at each side in the vertical direction towards the ground. The position of the side frame-door mechanisms was assessed as the deflection point [15]. The durability of the structure affects the crashworthiness for cars, because of the deformation of the body under dynamic loading. Hence, it may also influence the New Car Assessment Program (NCAP) crash rating for the car.

The simulated values for static bending and torsion were 10395 N/mm and 5483.53 Nm/deg, respectively. These values were subsequently evaluated in comparison to the values given by the manufacturers which were 10663 N/mm for torsion stiffness and 5421.76 Nm/deg for bending stiffness. The difference between the values, which were less than 3%, was because of the change in the elements order when different solvers were utilized [16]. The model was updated, smoothed, and cleaned from the original provided by the manufacturer to ensure the compatibility in terms of the solver. Therefore, the results from the model used for this research are slightly different from the manufacturer's though still acceptable.

Modal analysis was then done to find the natural frequency for the first torsion and bending modes under free-free boundary condition. The mass can also be calculated from the model to be used for the optimization analysis. A number of natural frequency values were taken from the modal analysis where the modes were selected based on the shape formed by the displacement of the elements.

**Figure 4:**
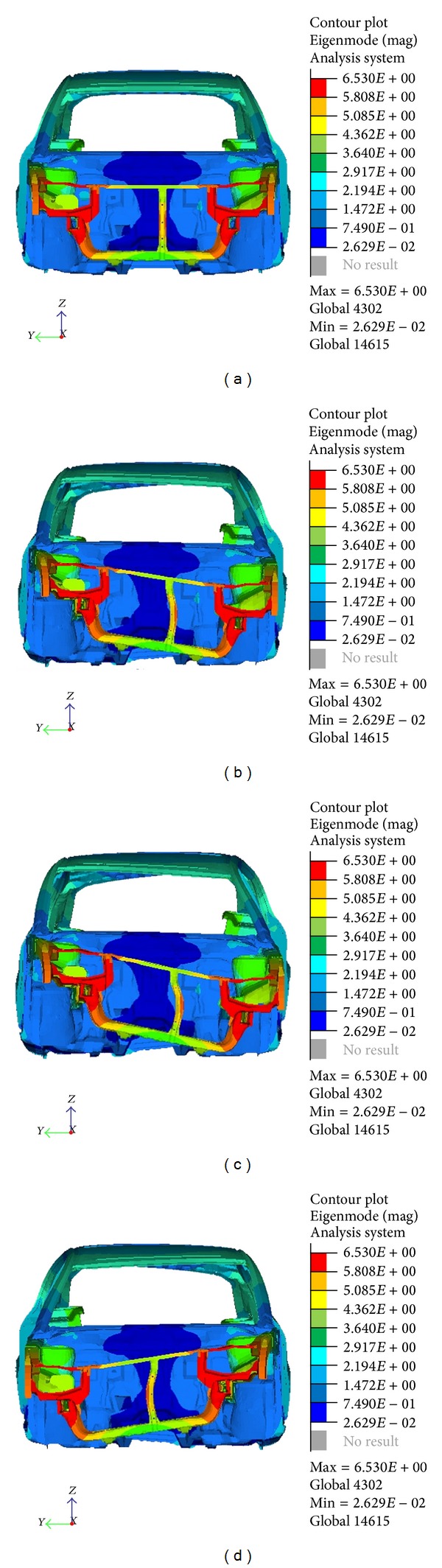
Torsion mode at 38.4 Hz.

**Figure 5:**
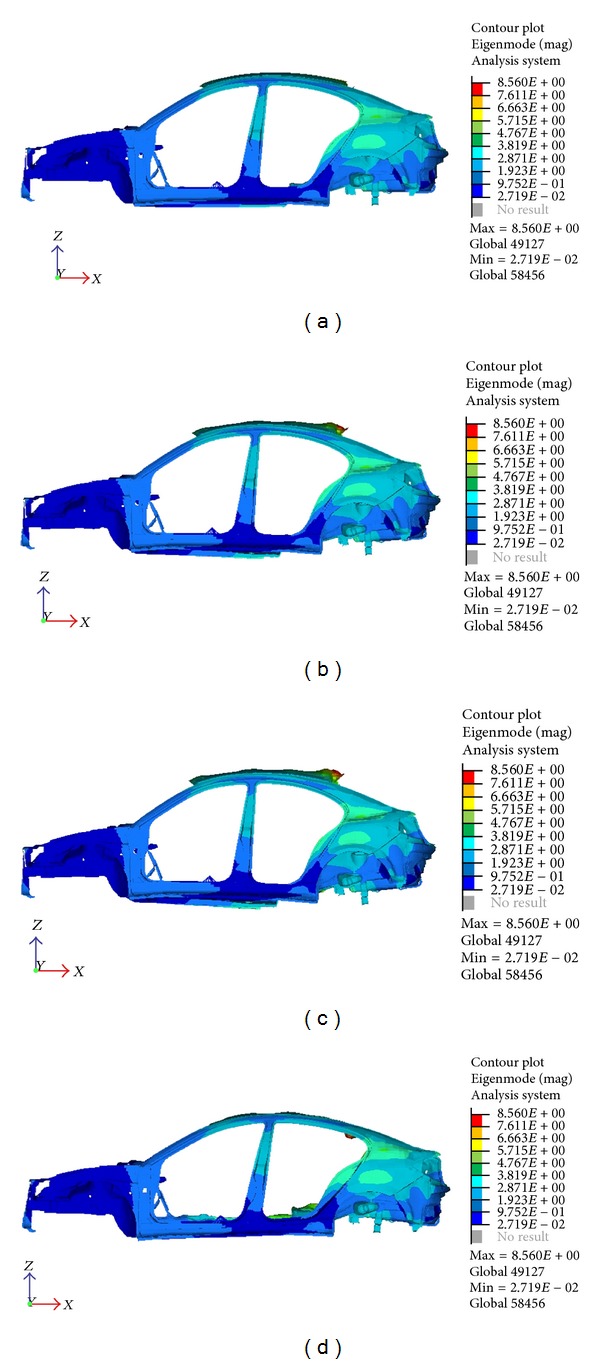
Bending mode at 51.5 Hz.

Figures 4 and 5 show the results from the analysis. The modes are distinguished by the deflection shape of the structure. The natural frequency is found to be significantly below the target value. Therefore, structural optimization will be applied to the model to increase the frequency for both modes by pinpointing the locations that need reinforcement using topology optimization.

## 3. Structural Optimization

Mathematically, structural optimization can be formulated by identifying the design variables, objective function, and constraints of the study. Basically, the general optimization problem [17] can be expressed with the following for a single objective function:

(1) $$\begin{array}{c} \text{Minimize}\;f\left(x\right)\\ \text{such}\;\text{that}\;g_{j}\left(x\right)\geq 0,\;j=1,\ldots ,n_{g},\\ h_{k}\left(x\right)=0,\;k=1,\ldots ,n_{e}, \end{array}$$

where *x* denotes a vector of the design variables. *h*_*k*_(*x*) denotes the equality constraint which is used when a constraint is set at a specific value. *g*_*j*_(*x*) is the inequality constraint and is used when the constraint needs to be within a certain limit. The inequality constraints are introduced in this study, where the natural frequency of certain modes needs to be increased to a specified value.

### 3.1. Topology Optimization

Under topology optimization, the material element density was set between 0 and 1 for all the elements by describing the elements as being void or solid, respectively [18]. The stiffness of the material would be assumed to change linearly according to the density of the elements. In general, optimal solution would result in intermediate density areas in the structural domain. Such solution would cause solution difficulties [19] such as complex material distribution of a given topology. The technique used to relax the problem uses continuous variables introducing penalization to force intermediate values close to the discrete values such as using solid isotropic material penalization model [20]. This model uses penalization technique by expressing the elements using “power law representation of the elasticity properties” as

(2) $$\begin{array}{c} \underline{K}\left(\rho \right)=\rho^{p}K, \end{array}$$

where $\underline{K}$ and *K* represent the penalized and real stiffness matrix of the elements, respectively. The *ρ* is the density and *p* is the penalty factor which is greater than 1. The material density of the elements *ρ*_MAT_ was calculated by subtracting the void area from the design such as the shell elements with *a* and *b* as the void sizes

(3) $$\begin{array}{c} \rho_{\text{MAT}}=1-\left(1-a\right)\left(1-b\right). \end{array}$$

The material density was used as the density design variable where the effective material property is linearly dependent on the element density. Therefore, a set value of a particular element density can be set as a benchmark to find which equates to the most alterations.

The topology optimization was conducted using Altair-Hyperworks/Optistruct with the thickness of the components as the design variable. The thickness is set to be able to increase until 4 mm from its original thickness. The component thickness was selected as the design variable, because the standard sheet metal used to reinforce body panel is between 0.2 mm and 1 mm [21] and the original maximum thickness is 3 mm. The responses used are the natural frequency for the two modes and the mass of the whole structure. The natural frequencies are then set as the constraints and the mass is set as the objective.

Referring to (1), the objective *f*(*x*) was set to minimize the mass of the whole BIW structure of the car. The optimization would be confined by the inequality constraints of the frequency of the modes which are to be greater than 40 and 60 for first torsion and bending modes, respectively. The design variable is the thickness of the shell elements where the value of 0 represents the original thickness and the value of 1 represents 4 mm of thickness.

**Figure 6:**
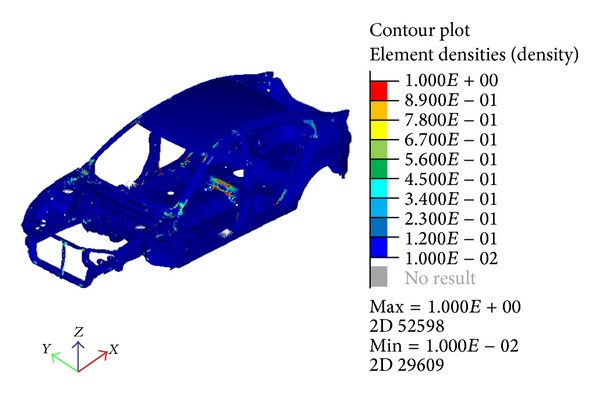
Optimization result by element density.

The result after the optimization (Figure 6) shows the locations where reinforcements are needed. Using the contour of the result as a reference, spots in red show the locations where changes in the components would result in significant improvement to the natural frequency. Spots in blue locate the region where no changes are necessary. A level set factor of 0.5 was used for the element density in the beginning. This means that only the elements that have a larger density than 0.5 would be chosen as elements for modification. A very small portion, 4.87% of the total elements, would need to be modified to satisfy the criteria.

With the modifications done using the suggestion from the optimization, both the frequency for torsion and bending modes were increased up to the proposed value. Furthermore, note that there is also an increase in the volume and mass of the structure, though it is of minimum value (Table 2). The extra load is the result of attempting to increase the value of the frequency without presenting any significant changes in the design.

The static test is conducted again on the optimized structure to predict the behavior of the body for the other factors. In retrospect, the added mass is not a welcoming solution to improve a BIW structure. The extra weight will affect the overall performance of the car. Therefore, a more thorough topology optimization may be of interest when finding the best arrangement for the structure.

One of the possible ways for optimization is performed by changing the maximum thickness of the new design. The use of 4 mm as the final thickness may not be the most ideal approximation for the optimization. The use of a smaller value may induce a change in the position of the reinforcement and may also reduce the added mass while still achieving the aim. The new value would still need to be above the largest original thickness of the components which is 3 mm, so the values of 3.2 mm and 3.5 mm are proposed for the new variable.

**Figure 7:**
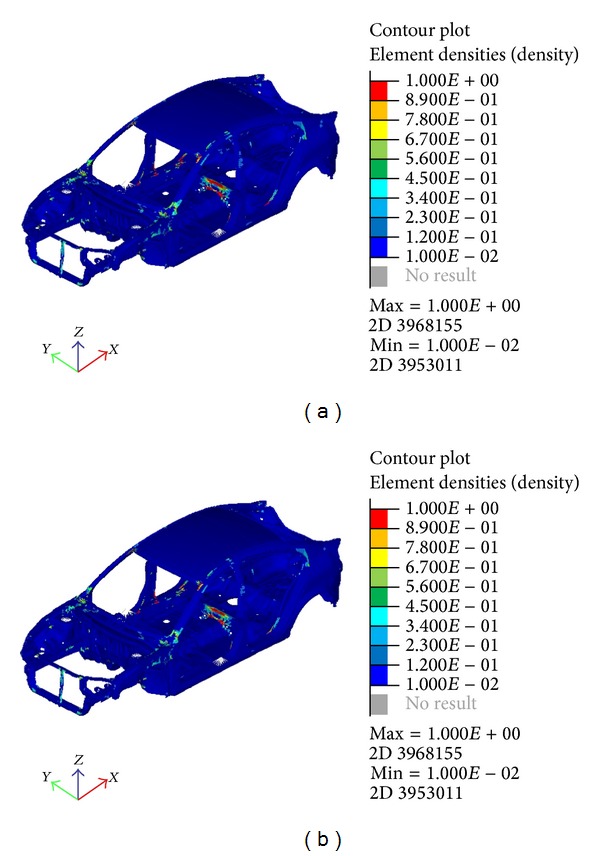
Optimization result using 3.2 mm (a) and 3.5 mm (b) maximum thickness variable.

By varying the thickness, the result of the optimization is also changed as shown in Figure 7 and Table 3, though the locations of improvement are still mainly the same. To satisfy the design criteria, 6.25% of the elements need to be changed for the thickness of 3.2 mm which is 5.55% for 3.5 mm thickness model. The same set factor of the element density was used in the previous optimization. This means that, with a smaller value for the maximum thickness, more elements would require changes to achieve the optimization objective. However, the comparison does show a much better result of the optimization with lower added mass and a fairly close frequency for both modes. This explains that the thickness of the reinforcement does not need to be large to achieve a good result. Further thorough investigation is needed to find the optimal thickness for the optimization to lower the added mass.

### 3.2. Size Optimization

After completing the topology optimization on the structure, the scattered elements that will be used for reinforcement can further improve the structure. The behavior of structural elements such as shells, beams, rods, springs, and concentrated masses is defined by input parameters, such as shell thickness, cross-sectional properties, and stiffness. These input parameters can be modified in a size optimization process. The property of the material is not the design variable itself but is defined as a function of the design variable. It is defined by a design-variable-to-property relationship which is a linear combination of design variables such that

(4) $$\begin{array}{c} p=C_{0}+\sum {\text{DV}}_{i}\cdot C_{i}, \end{array}$$

where *p* is the property to be optimized and *C*_*i*_ are linear factors of the design variable, DV_*i*_. However, for simple gage optimization of shell structures, the relationship changes where gage thickness will be identical to the design variable.

The same objective function was implemented for the optimization as shown in (1). Initially, every component of the structure is considered as the designable property and therefore this process uses the same criteria as in Table 1. Since the structure comprises 156 different components, the optimization process uses sizable resource to complete, because each part would need to be adjusted with different design-variable-to-property relationship. Using this approach, the natural frequency of both modes increased successfully, but with a mass increase of 335.1 kg. Although the result shows a very reasonable consequence of the frequencies and the total mass, the substantial changes which involve almost all components indicate that major modifications and alterations are required in the original structure. This in turn would also shift the capability of the structure in another aspect such as crashworthiness.

Consequently, a method to avert this was to make use of the result from the topology optimization. The optimization criteria used are similar to the criteria set for the topology optimization previously except for the design variable. Rather than using all the elements as the design variable, only the elements in the components that need reinforcements in the topology optimization were used. As a result, the number of elements or components that will be modified is significantly less than before.

The objective function *f*(*x*) was set to minimize the mass of the whole structure under the constraints of the frequency of the first torsion and bending modes as shown in Table 4. The thickness of the particular components will be the design variable with the original thickness as the initial value and 3.2 mm or 4 mm as the maximum thickness. The two different thicknesses used for the maximum thickness will assist in distinguishing the better combination of the two optimizations.

The result of the optimization using a level set factor of 0.5 from the topology optimization is shown in Table 5 where some components with intermediate elements were also chosen as the design variable. Therefore, the chosen components are moderately higher than the case where the selected elements have element density closer to 1. There are 54 and 46 components that will be adjusted for the 3.2 mm and 4 mm thickness optimization, respectively. After changing the thickness of the components, the frequencies of both modes were increased to 40 and 60 Hz as intended. The mass was also increased to 335.6 kg for the 3.2 mm and 337.4 kg for the 4 mm. These indicate that the size optimization using 0.5 element density will change the thickness of a considerable number of components which in turn will significantly alter the behavior of the whole structure. For that reason, an optimization is performed using only the components where the elements have a density closer to 1.

Size optimization was done again using a level set factor of 0.9 as the element density. This involves only 28 components for the 3.2 mm and 22 for the 4 mm which is significantly less than the previous approach. From Table 5, it is shown that the mass of the new structure has increased significantly more than the structure optimization using 0.5 for element density. While the objective of increasing the frequencies was achieved, the large increase in mass is too unfavorable in the construction of a vehicle body.

Using the result from all of the different approaches for size optimization, it is shown that the added mass is notably higher than from the topology optimization. This is because it not only changes the elements intended for improving the stiffness but also changes the entire component related to the elements. By doing this, it may have increased the added mass but it will increase its manufacturing feasibility. By having a constant thickness throughout the parts, the same manufacturing process can be used to create the component without requiring any further process to add reinforcement to the initial component.

### 3.3. Static Test of Optimized Models

Both static tests were performed again for the recent models to check the validity and quality of the optimization methods and results. The models were examined on their torsion and bending static stiffness test as previously executed for the original model.

In Table 6, the new models that aimed to improve the dynamic property of the structure do not show an increase in the static stiffness. Two out of the three approaches resulted in a decrease of static stiffness pertaining to either bending or torsion stiffness. The only approach that shows an increase in both is the model undergoing topology optimization with maximum thickness of 4 mm. The approach demonstrates that the optimization may improve the dynamic characteristic while reducing the static stiffness. It is therefore recommended to check if the improvement impacts other related properties and factors.

### 3.4. Combination of Size and Topology Optimization

By examining the topology optimization results from Figures 6 and 7, it is shown that there exist components that need considerable change or modification. Some even show that a lot of the elements need to be changed in a single component. For that reason, these components may establish a better solution when only experiencing size optimization rather than the topology. In this case, the problem such as stress concentration from the varying thickness can be avoided.

**Figure 8:**
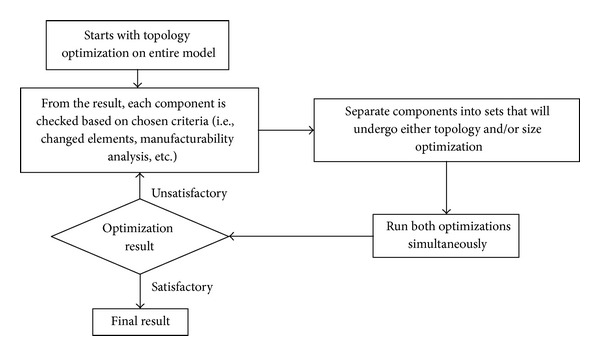
Combination of size and topology optimization strategy.

Therefore, a combination of optimization processes is proposed where the components are distributed between topology and size optimization. The strategy utilized to combine the optimization processes is shown in Figure 8. The set components were divided by calculating the percentage of the changed element in a single component after topology optimization. Hence, components with a large number of changed elements will only undergo size optimization. There may be better assumption methods to group the components such as manufacturability analysis [22].

As a trial run, a setup of the topology optimization with maximum thickness of 4 mm was used as the basis. From the topology run, the components were separated into two categories to be used in two different optimization processes. The components with the most practical changes were Comp 5012021, Comp 5018020, and Comp 5018022, where these three will only go through size optimization without any change to the design.

**Figure 9:**
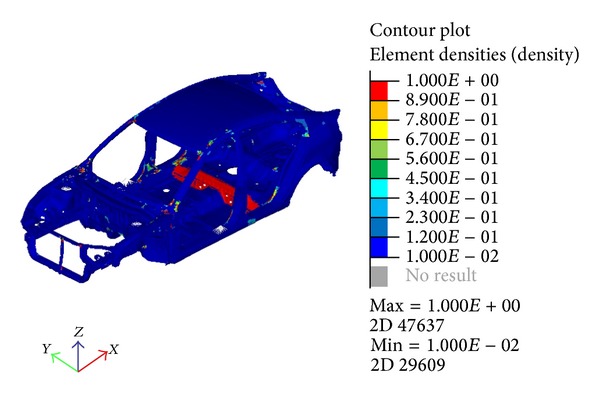
Combination of size and topology optimization result.

Figure 9 shows the result from the optimization where both size and topology were used. The new model illustrates the points where reinforcement is needed except for the three components undergoing size optimization. The changes of thickness for the three components are as follows:

- Comp 5012021: 0.75 mm to 2.544 mm,
- Comp 5018020: 1.2 mm to 1.99 mm,
- Comp 5018022: 1.5 mm to 0.5297 mm.

The new natural frequencies for torsion mode and bending mode were successfully increased to satisfy the objective with value of 42 Hz and 60 Hz, respectively. The whole mass also increased by about 7.5% from 275 to 295.7 kg. This reveals that the method of combining both optimizations is acceptable as it does satisfy the objective while maintaining the low mass. Then, the static stiffness test was also conducted to show feasibility of the approach. The result for bending stiffness shows an increase of 4.16% to 10827.2 N/mm while torsion stiffness shows an increase of 0.25% to 5497.23 Nm/deg in terms of stiffness. Thus, by combining the optimization methods, the model can be improved in regard to its dynamic characteristics.

## 4. Discussions

It is shown that reasonable improvements can be achieved for the torsion and bending modes natural frequencies. The application of simultaneous optimization on both modes was found to be efficient in obtaining the solutions that satisfy both vibration modes. Additionally, the amount of changes needed was minimized, because the locations requiring improvements were influenced by either one or both modes. So, the required changes to the design depend on the optimization process. It is evident that the topology optimization using element thickness as the variable requires minimum mass to reinforce the structure which in turn increases the bending and torsion stiffness. The topology optimization with different maximum thicknesses has shown significant improvement in the dynamic characteristics of the structure by a small increase in mass. However, the modifications needed to reinforce the components would introduce other problems such as manufacturability and stress concentrations. It is shown that for all topology optimization approaches, there are common components that need reinforcement to increase the natural frequency. These components can be used as the basis for the improvement of the whole structure.

So, size optimization was used to overcome the problem by realizing the optimum change in the thickness of specific components in whole rather than adding reinforcements. Nevertheless, this approach would yield a larger increase in mass compared to the topology optimization result.

Therefore, a practical and reasonable balance between both optimizations is needed to find the best modifications of the components to achieve the objective. In conclusion, a combination of topology and size optimizations is found to be a much better alternative to improve the dynamic characteristics of a BIW structure while allowing minimum added mass.

### 4.1. Common Components

**Figure 10:**
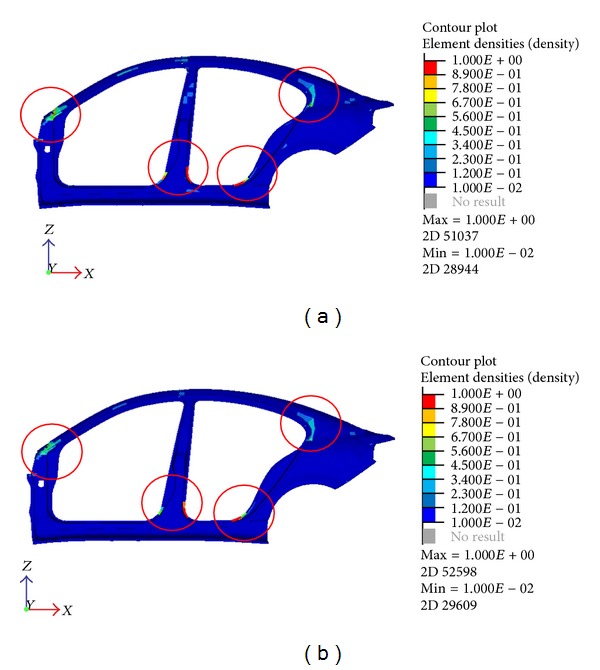
Highlight of reinforcement areas on outer quarter panel for 4 mm and 3.2 mm.

Some components from the result of the topology optimization show that the placement of the reinforcement is miniscule compared to the size of the component such as the outer quarter panel (Figure 10). Thus, by applying size optimization and increasing the thickness of the entire component, it may result in an unnecessary and additional mass. So, it is wise to consider the implication of either using size optimization on certain components or just reinforcing the components at a specific area according to the topology optimization as shown in Section 3.4.

However, it is shown that the modification of thickness at specific areas of the component results in a stress concentration in those areas [23]. The concentration may induce other problems like fatigue crack initiation [24]. One possible way to alleviate stress concentration is to change the thickness gradually. A smaller fraction of the maximum thickness should also decrease the concentration. Another method of preventing stress concentration is to use additional reinforcing material instead of using components with varying thickness. These additional materials may be welded or bolted to the existing structure, although this may create other problems such as weak welds [25] where cracks may initialize. Hence, a thorough investigation is needed prior to any application.

**Figure 11:**
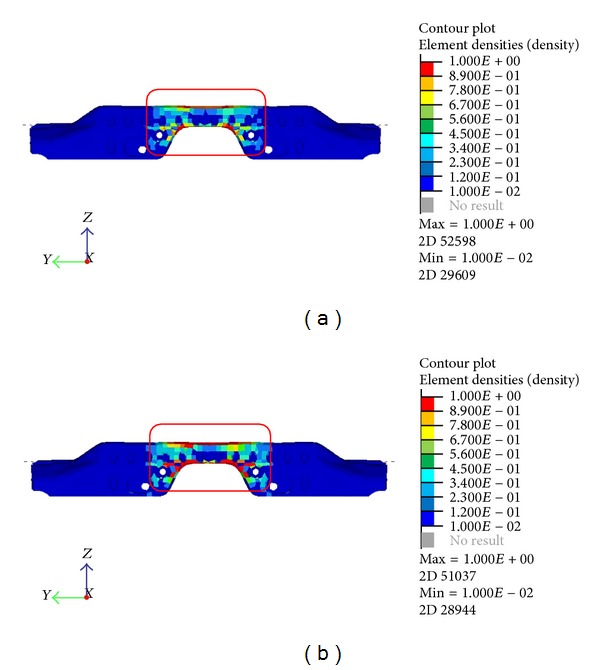
Highlight of reinforcement areas on rear seat center member and rear floor extension 4 mm and 3.2 mm.

On the other hand, there are certain parts in the area of fortification which covers the rear seat center member and the rear floor extension. This area covers almost one-third of the whole components as shown in Figure 11. Hence, to improve such components, size optimization would be the most suitable choice. Therefore, to achieve the objective while still maintaining the practicability of a BIW of a car, the best method would incorporate the result from both topology and size optimization following the effectiveness of the modification.

### 4.2. Comparison of All Results

After collecting all the results and outcome from different approaches of optimization, a comparison in terms of the new natural frequency, mass, static stiffness, and manufacturability concern was conducted. The assessment would express the varying benefits and drawbacks between different approaches and should be chosen depending on the need of the structure.

From the previous sections, it was revealed that all of the optimization approaches were managed to improve the dynamic characteristics of the structure to the specified value. Both natural frequencies of the torsion and bending modes were increased to exceed the target values which were 40 Hz and 60 Hz, respectively. In spite of this, these several diverse approaches were also employed to determine the effect of the process on other factors.  Table 7 shows the values of the mass and static stiffness of the new models obtained from the optimization processes.

The result from topology optimization only shows that the new model is ideal because of minimal mass addition with an increase in stiffness. The only drawback from topology optimization was about the minor area of changes of thickness in the components that may induce stress concentration as explained in Section 4.1. To reduce the aforementioned problem, size optimization was used to completely change the thickness of the whole component rather than just part of it. The result from the size optimization shows that the mass increase is very significant and the bending stiffness was also compromised because of it. For these reasons, a combination approach where both topology and size optimization were utilized was introduced. This new method resulted in a model with low added mass and still satisfactory static stiffness. It also decreased the amount of small area changes needed to the components of the structure.

## 5. Conclusion

The objective of increasing the frequency of both torsion and bending modes to specified values was achieved by using the proposed method. The approach of using structural optimization to improve both bending and torsion modes simultaneously was found feasible by setting constraints for the predetermined vibration modes. The improvement was done by either adding reinforcing material or changing the thickness of the components. Moreover, the changes of the BIW from the original were minimized by adjusting the objective of the optimization to minimize the whole mass. The structure after the modification also shows change in the static stiffness in a small amount. Therefore, the topology or size optimization or a combination yields a BIW that produces a more balanced solution comprising higher natural frequencies for the respective modes with minimal drawbacks such as weight additions, manufacturability, and stress concentrations.

## Figures and Tables

**Table 1:** Topology optimization criteria settings.

Variable	Thickness of all shell elements, *T* (original thickness < *T* < 4 mm)
Constraints	First torsion mode >40 Hz, first bending mode >60 Hz

Objective	Minimize mass

**Table 2:** Result comparison of original and optimized model.

Parameter	Model		Difference	%
	Original	Optimized		
Torsion (Hz)	38.4	42.6	4.2	10.9
Bending (Hz)	51.5	60.0	8.5	16.5
Mass (kg)	275.0	294.0	19.0	6.9

**Table 3:** Result comparison of using 3.2 mm and 3.5 mm thickness variable.

Max thickness	3.2 mm		3.5 mm	
Parameters	New frequency	% difference from original	New frequency	% difference from original
Torsion (Hz)	42.5	10.7	42.6	10.9
Bending (Hz)	59.8	16.1	59.6	15.7
Mass (kg)	291.1	5.85	291.8	6.11

**Table 4:** Criteria of size optimization depending on topology optimization.

Variable	Thickness of shell elements selected from topology optimization, *T* (original thickness < *T* < 3.2 mm or 4 mm)
Constraints	First torsion mode >40 Hz, First bending mode >60 Hz

Objective	Minimize mass

**Table 5:** Size optimization result.

Max thickness	3.2 mm		4 mm	
Element density	New mass (kg)	% difference from original	New mass (kg)	% difference from original
Factor of 0.5	335.6	22.04	337.4	22.7
Factor of 0.9	424.6	54.4	415.4	51.05

**Table 6:** Static test of models from optimization result.

Optimization method	Static bending test		Static torsion test	
	Result (N/mm)	% change	Result (Nm/deg)	% change
Topology—3.2 mm max thickness	11989.0	15.33	4821.67	−12.07
Topology—4 mm max thickness	10860.1	4.47	5916.68	7.9
Size—all components	10073.5	−3.09	6230.19	13.6

**Table 7:** Mass and static stiffness results from each optimization approach.

Approach	Mass		Static stiffness			
			Bending (N/mm)		Torsion (Nm/deg)	
	Value	% change	Value	% change	Value	% change
Original	275	—	10395	—	5483.5	—
Topology—4 mm thickness	294	6.9	10860	4.47	5916.7	7.9
Size—all components	335.1	21.9	10074	−3.09	6230.2	13.6
Combination	295.9	7.45	10827	4.16	5497.2	0.25

## References

[1] Bower AF (2010). *Applied Mechanics of Solids*.

[2] Thorby D (2008). *Structural Dyncamics and Vibration in Practice: An Engineering Handbook*.

[3] Xu CS, Yi HH, Huang CX Experimental study of vehicle modelling and ride comfort simulation based on the topology structure analysing.

[4] Lu J, DePoyster M (2002). Multiobjective optimal suspension control to achieve integrated ride and handling performance. *IEEE Transactions on Control Systems Technology*.

[5] Billah KY, Scanlan RH (1991). Resonance, tacoma narrows bridge failure, and undergraduate physics textbooks. *American Journal of Physics*.

[6] Parrish A, Rais-Rohani M, Najafi A (2012). Crashworthiness optimisation of vehicle structures with magnesium alloy parts. *International Journal of Crashworthiness*.

[7] Untaroiu CD, Shin J, Crandall JR (2007). A design optimization approach of vehicle hood for pedestrian protection. *International Journal of Crashworthiness*.

[8] Zheng ZC, Guo D, Zhang Y, Hou Z (2001). Dynamic analysis of large-scale flexible systems for free-free space structures. *Philosophical Transactions of the Royal Society A: Mathematical, Physical and Engineering Sciences*.

[9] Kim K, Kim C (2005). A study on the body attachment stiffness for the road noise. *Journal of Mechanical Science and Technology*.

[10] Sugahara Y, Kazato A, Koganei R, Sampei M, Nakaura S (2009). Suppression of vertical bending and rigid-body-mode vibration in railway vehicle car body by primary and secondary suspension control: results of simulations and running tests using Shinkansen vehicle. *Proceedings of the Institution of Mechanical Engineers F: Journal of Rail and Rapid Transit*.

[11] Jang G, Choi Y, Choi G (2008). Discrete thickness optimization of an automobile body by using the continuous-variable-based method. *Journal of Mechanical Science and Technology*.

[12] Vanderplaats GN (1984). *Numerical Optimization Techniques for Engineering Design: With Applications*.

[13] Wang L, Basu PK, Leiva JP (2004). Automobile body reinforcement by finite element optimization. *Finite Elements in Analysis and Design*.

[14] Blakey K MSC/NASTRAN basic dynamic analysis user's guide: version 68.

[15] Happian-Smith J (2002). *An Introduction to Modern Vehicle Design*.

[16] Entwistle KM (2001). *Basic Principles of the Finite Element Method*.

[17] Haftka RT, Gürdal Z, Kamat MP (1990). *Elements of Structural Optimization*.

[18] Bendsøe MP, Sigmund O (2003). *Topology Optimization: Theory, Methods, and Applications*.

[19] Belblidia F, Hinton E (2002). Fully integrated design optimization of plate structures. *Finite Elements in Analysis and Design*.

[20] Bendsøe MP, Sigmund O (1999). Material interpolation schemes in topology optimization. *Archive of Applied Mechanics*.

[21] Duffy JE (2009). *Auto Body Repair Technology*.

[22] Gupta SK, Regli WC, Das D, Nau DS (1997). Automated manufacturability analysis: a survey. *Research in Engineering Design*.

[23] Timoshenko S, Goodier JN (1969). *Theory of Elasticity*.

[24] Huynh J, Molent L, Barter S (2008). Experimentally derived crack growth models for different stress concentration factors. *International Journal of Fatigue*.

[25] Gaul H, Brauser S, Weber G, Rethmeier M (2012). Methods to obtain weld discontinuities in spot-welded joints made of advanced high-strength steels. *Welding in the World*.

